# Material History, Historied Materials and the Question of Epistemic Freedom in Ghana’s Medical Schools

**DOI:** 10.1093/shm/hkad039

**Published:** 2023-09-06

**Authors:** John Nott

**Keywords:** medical education, material history, Ghana, imperialism, epistemology

## Abstract

Medical schools rely on a wide range of tools, technologies and materials for their teaching, on books, and bodies, and on the buildings which house them. This article considers the histories of this material culture in the three oldest medical schools operating in Ghana today. Borrowing theoretical concepts from Science and Technology Studies, medical anthropology and postcolonial political economy, this article takes that the material culture of modern medical education often binds contemporary pedagogy to outdated ideas and faraway places. The agential, proselytising nature of these historied materials agitates against the localisation of biomedicine and contributes to a distracting scientific imaginary which remains centred around historical, often imperial centres of knowledge production in Europe and North America.

The pathology museum at the University of Ghana Medical School (UGMS) in Accra, the oldest medical faculty in the country, has been largely closed to students for nearly a decade now.^1^ Named for William Neizer Laing, the pioneering Ghanaian pathologist and later university administrator, the W.N. Laing Pathology Museum opened in 1970 and soon grew into one of the foremost collections of pathological specimens on the continent. The collection is still maintained, up to a point, but many of the wet specimens are completely desiccated; the formalin which fixes these old presentations of disease is always evaporating but is not always available. The relative absence of the expensive, imported materials which were necessary to keep the museum open—from hermetically sealed containers to laboratory chemicals—has been compounded by a pervasive, international shift away from museum collections in medical pedagogy.^2^ In Accra, as elsewhere, preserved specimens have been displaced by teaching materials which are more able to cater to ever-growing class sizes, and which are also less costly and time-consuming to prepare and maintain. The spread of first audio-visual and then digital technologies—produced primarily in Europe and North America—precipitated the deterioration of the Laing collection and the erasure of the localised pathologies which it helps to preserve (Figure 1). The story of the Laing Museum, its development and then decline, is part of a longer history of medical epistemology, one in which the material culture of education plays an important, if underexplored, role.

**Fig. 1.**
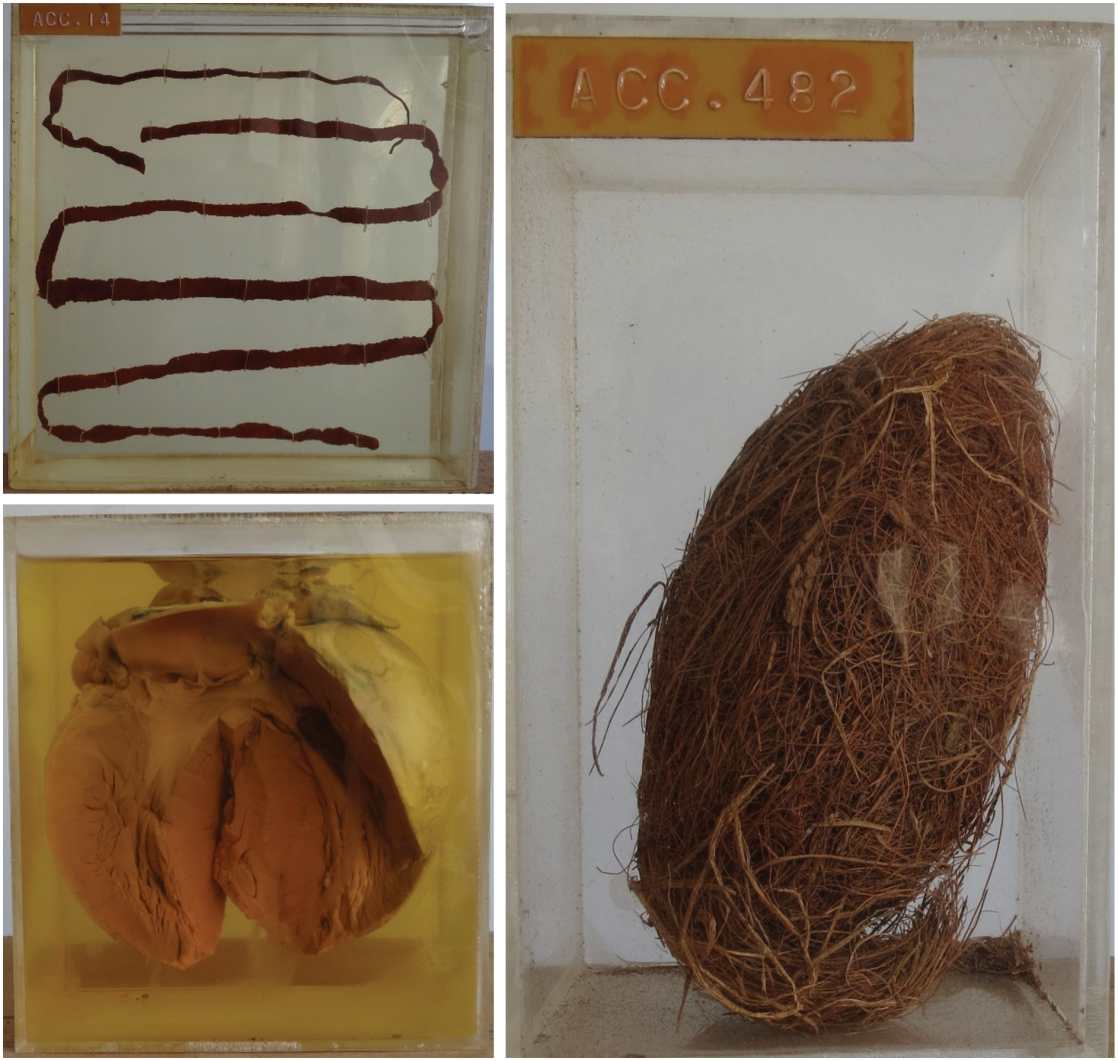
Potted specimens at the Laing Museum. Clockwise from top left: pot containing a tapeworm, now rarely seen in Ghana; pot containing a bezoar composed of coconut husk, from a patient with a psychiatric disorder; pot containing a heart showing hypertrophic cardiomyopathy, at the time of preservation the condition was uncommon in Ghana. Courtesy of Robert Kumoji.

The history of biomedicine in Africa, as everywhere, is also a history of the tools and technologies used in the education of its practitioners. Ideas and practices are spread and shared, reified and reproduced alongside the textbooks, anatomical models and preserved specimens which populate medical schools. Despite their necessity and ubiquity, the circulation of these materials and has received little academic attention. Several histories have detailed the development of the medical profession in Ghana, and other parts of the continent, but the material, practical and epistemic history of medical education has been largely overlooked.^3^ Addressing this absence elsewhere in the medical humanities, Claire Wendland’s argument, developed out of ethnographic research in a Malawian medical school, takes ‘that technologies can be potent actors even when they are materially absent’.^4^ Here Wendland emphasises the tension between the ‘abstract presence and material absence’ of a spectrum of clinical technologies—from gene assays and magnetic resonance angiograms, through to simpler technologies, radiation therapy, dialysis or artificial ventilation. The presence of these technologies in contemporary curricula directly contradicts their absence from the wards of the teaching hospital.^5^ This article explores the origins of these contradictions since, although relative access to diagnostic and therapeutic technologies clearly plays on the minds of young clinicians, there is an ecology of educative materials which stokes students’ imaginations. These imaginaries have consequences. Conceptualisations of what medicine could or should be has been said to drive the outmigration of physicians throughout Africa.^6^ A 1999 study found that more than 60 per cent of doctors trained at UGMS between 1985 and 1994 had left the country, the vast majority leaving for the UK or the USA.^7^ A 2012 survey of medical students across all universities found that more than half still intended to move abroad following the completion of their housemanships.^8^

Since Ghana’s independence from British imperial government in 1957, seven medical faculties been established—five public and two private—all of which provide excellent medical training, and which have all contributed to huge increases in terms of educational capacity and numbers of practicing physicians. These physicians have, in turn, fostered a wealth of academic research and a concentration of practical expertise which has worked to localise the *production* of biomedical science. Although conscious of these histories, this article explores material resistance to such change, taking instead that the material culture of medical education often instead works towards the *reproduction* of a generalised historical episteme, binding contemporary practice to old ideas and faraway places.^9^ While the traditional, discursive approach favoured by cultural historians takes that artefacts are simply vessels for human culture, Science and Technology Studies (STS) research emphasises the ways that culture is shaped and constrained by materiality. Material culture should, therefore, be seen as an agential force within science, one which encourages specific ideas and particular practices.^10^ Although the weight of material history affects and directs the use of all forms of technology, the proselytising nature of educative, epistemic materials makes their original inscription especially difficult to escape. Textbooks, for instance, detail ‘an already articulated set of problems, data, and theory’ and, in this respect, argue for ‘the particular set of paradigms to which the scientific community is committed’.^11^ These paradigms are necessarily historical and, in the specific case of Ghanaian medical education, the endurance of colonial-era materialities, as well as the continued reliance on imported epistemic materials, might be understood as a form of ‘imperial debris’, one which contributes to the reproduction of ‘cognitive empires’ and Western authority over biomedical science.^12^ This article suggests that efforts to resist the gravity of historic and often imperial centres of knowledge—to localise or even decolonise biomedical epistemology—are similarly impeded by the historied material culture of medical education.^13^

Since the 1960s, the decolonisation of African universities has primarily focused on the Africanisation of personnel, rather than curricula, pedagogical structures or epistemologies.^14^ A recent reinvigoration of this scholarship has renewed focus on these more fundamental aspects of university instruction.^15^ Medical schools have, however, rarely been a primary locus of radical epistemic reform, nor are they currently considered a prime locus of contemporary coloniality. This is partly due to a historical tension inherent in any decolonisation of medicine. While decoloniality proffers liberatory epistemologies that seek to ‘de-link from the tyranny of abstract universals’, the biological sciences and biomedical best practice is based on the assumption that the human body is ‘universal and amenable to intervention through standardized approaches to medical management and care’.^16^ Any suggestion that biological difference might reflect in human populations was widely rejected following the Second World War, and the horrors perpetrated in the name of eugenics. In recent years, however, biomedical universalism has begun to soften. Today, medical anthropologists emphasise the ontological instability, situatedness, and ‘fluidity’ of human biology and biomedical practice.^17^ Despite this, the technologies used to teach medicine deviate towards assured and relatively fixed understandings of bodies, diseases and cures. These tools are more akin to ‘immutable mobiles’, objects which, in Bruno Latour’s initial explanation of the concept, centred on printed text and offered ‘translation without corruption’.^18^ Although biomedicine is never stable, epistemic technologies often suggest that it could be, that it has been or that it might be somewhere else. This does not mean to say that they cannot be softened, even to the point of fluidity, but that any such ‘de-scription’ has its own cost in terms of time, or money, or both.^19^ In postcolonial contexts, economic constraints affecting tertiary education means that this is often much less forthcoming.

Historians and philosophers of science have long emphasised the role of textbooks in the reproduction of science, as arbiters of specific paradigms and abstract ideas. Yet they also have an affective and variable material quality; they might be new or old, fresh or tattered, they derive from particular times and specific places, and this also affects their material qualities. In this respect, textbooks are one part of a broad material culture of education. Embracing everything from the textbooks, plastic models and cadavers which populate medical faculties, through to the buildings which house them, this article attempts an historicisation of the STS research which explores how medical practice develops in conjunction with the material environment.^20^ Despite their importance to the history and contemporary practice of medicine, these materials are often not well-attended to in the archival record, nor is the slow process of learning ideas and skills well-remembered. As such, this article attempts a historical ‘implosion’ of the material culture of medical education in the present, working backwards through a range of oral, archival and ethnographic evidence in order to show the various, inconsistent historical threads which pull at the contemporary practice and epistemology.^21^ Here we primarily deal with the three oldest schools in Ghana: the University of Ghana (UGMS), in Accra; the Kwame Nkrumah University of Science and Technology (KNUST), in Kumasi; and the University for Development Studies (UDS), in Tamale. In three discrete sections, we move from the cloistered, colonial-era *buildings* which continue to dominate the landscape of postcolonial education; through the *books* which reproduce Western authority over ‘tropical’ medicine; and, finally, to the various *bodies* used in teaching. While the historied material culture of medical education tends to site scientific authority elsewhere, it is the knowing bodies of physicians and the known bodies of their patients which provides some reprieve. Yet, as the story of the Laing Museum suggests, the means to preserve and reproduce this knowledge often prove fragile.

## Buildings

The medical faculty at the University of Ghana was always supposed to be appended to the rest of the University, but it is only recently that a teaching hospital has been built on the main campus at Legon. Initially situated at the far edge of Accra, the city has since grown up around this sprawling, leafy, manicured site, somewhere which has remained spatially removed and materially distinct from the surrounding city. Separated from each other by wide, tree-lined boulevards, most of the buildings on the campus are in the classic colonial style—low-rise, with white-washed stone and red terracotta roofs. All of Ghana’s universities are products of distinct histories but, perhaps more than any other aspect of their material culture, it is their buildings and where they stand which belie the context of their construction. While textbooks, journals, manikins and preserved specimens might be replaced, removed or—as in the case of the Laing Museum—deteriorate over a relatively short space of time, buildings are often more resistant to change. An expansive reflection on the material environment of medical education must then also consider the spatial and architectural imposition which contributes to the experiential emplacement of learning.^22^ Although the ongoing agency of users, caretakers, cleaners and owners contributes to changes of use, deterioration and demolition, the ‘obdurate’ siting of teaching hospitals, dormitories, libraries and lecture theatres all form part of what Alice Street has termed the ‘affective infrastructure’ of biomedicine.^23^

As detailed in R.B. Bening’s seminal historical geographies of educational disparity, there is a spatial politics which has contributed to the siting of universities across Ghana.^24^ The campus at Legon and the teaching hospital at Korle Bu—arguably still Ghana’s most prestigious educational and medical institutions—are especially enduring material legacies of infrastructure developed in support of imperial ambition.^25^ In the 1940s, as Bening notes, many African voices advocated for the development of a university in the colony, but Legon was rarely their first choice. ‘It is in the midst of the non-African influences of the town of Accra’, worried one politician from Kumasi, ‘what the people of this country want is a university sited at such a place as will make the people of this country feel that it is a common property’.^26^ The British administration took a rather different view. The final 1946 report into the establishment of a university found that, ‘from the aesthetic point of view, there is everything to commend it’, including that its hilltop position meant that ‘the university buildings [would] be visible from a great distance’.^27^

Siting universities in commanding geographies and distinctly imperial spaces was a common aspect of education policy in British colonies across West Africa. Tim Livsey—whose research on infrastructures of higher education in Nigeria offers valuable context and comparison for the Ghanaian history—has described this as the ‘imperial frame’ of universities which started under empire.^28^ These schools, and their medical faculties, were intended to promote the development of a professional class along British lines. Prior to 1964, when UGMS accepted its first pre-clinical students, medical students were trained overseas; the majority receiving scholarships or privately funding study in British institutions. In a 1940 report on the awarding of medical scholarships, the Director of Colonial Scholars explained that the value of ‘[African] acquaintance with English life and institutions, [was] the friendships they form in this country, [and] their influence as leaders of native public opinion’.^29^ Colonial educationists were generally of the opinion that ‘students should be sent only to residential universities … in the United Kingdom [so] that they return with a higher appreciation of British life and civilisation’.^30^ For medical students, however, there was ‘constant difficulty in letting [students] have adequate clinical work, owing to differences which are entirely racial’.^31^ Some in the Colonial Office worried that such discrimination might ‘embitter’ a future elite against British government.^32^ The transposition of the material environment from Britain to West Africa provided a solution which would also transpose the ideals bound up in the British university; the Inness Report into the potential development of a West African medical school emphasised that residence at the College ‘must be regarded as an indispensable part of their professional training’.^33^ From its outset, however, medical education in colonial West Africa was designed to be vocational, geographically and epistemologically peripheral to the fuller, academic education provided in British universities.^34^ Any such school was to offer a ‘course of study of limited scope … and not to attempt to give, in the first instance, training in medicine to the professional standards recognised in Great Britain’.^35^ Graduates would not be granted license to practice outside of West Africa.

In the end, the Colonial Office chose Nigeria as the site for its West African medical school, with the Yaba College of Medicine opening just outside of Lagos in the 1930s. For the most part, Ghanaian medics carried on training in British schools. Following Ghanaian independence in 1957, however, President Kwame Nkrumah considered the internal training of doctors as an essential part of national self-determination.^36^ Between the late 1950s and early 1970s, several plans were made for the construction of an integrated medical school and teaching hospital on the Legon campus. These were to be modernist, high-rise buildings which, like the emergent nation, were based on the ideals of self-determination, ‘designed … [to] encourage patients to help themselves as soon as possible, as a therapeutic measure’.^37^ Later plans emphasised the novel architectural design and high-tech communications technologies which would help integrate teaching and treatment as part of a ‘continuous and harmonious’ system. Buildings were to be linked by covered walkways while telephone lines and closed-circuit television would link classrooms with clinical work.^38^ Yet, for myriad reasons, although all largely related to the cost of such a project, this school was never built.^39^ Instead, clinical education at UGMS was centred around Accra’s main public hospital at Korle Bu, with medical students ferried back and forth, between the hospital and their dormitories at Legon. In this respect, it was practical expediency and local contingencies which shaped the initial development of medical training in Ghana, rather than the prescriptive planning of colonial or postcolonial governments. Even so, the hasty development of a teaching hospital around Korle Bu, a centrepiece of imperial infrastructure, speaks to the obduracy of colonial-era spaces.

**Fig. 2.**
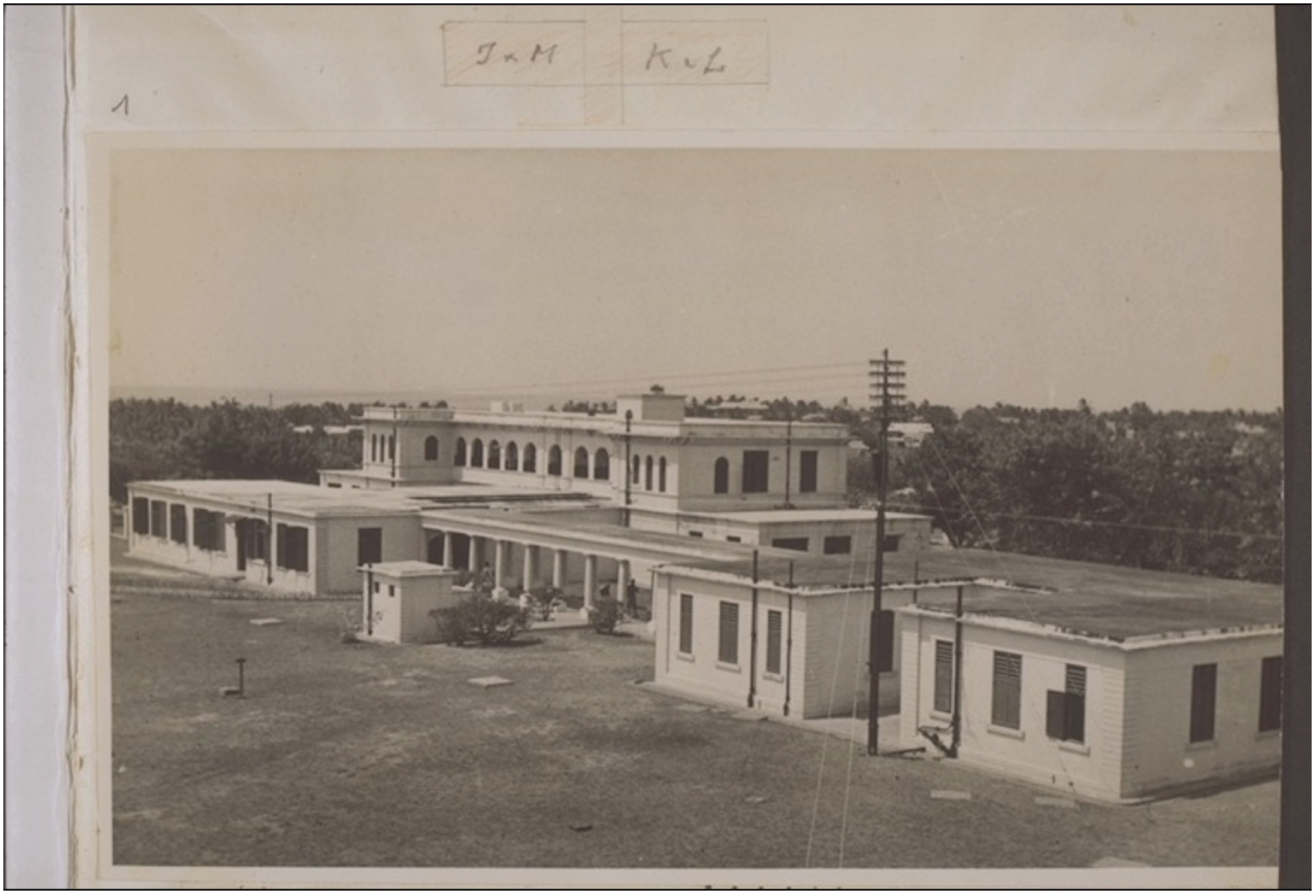
The outpatients’ department at Korle Bu Hospital, c. 1940s (West African Photographic Service, Accra). Courtesy of Basel Missionary Archive, BMA QD-30.102.0001. See also Roberts p. 210.

**Fig. 3.**
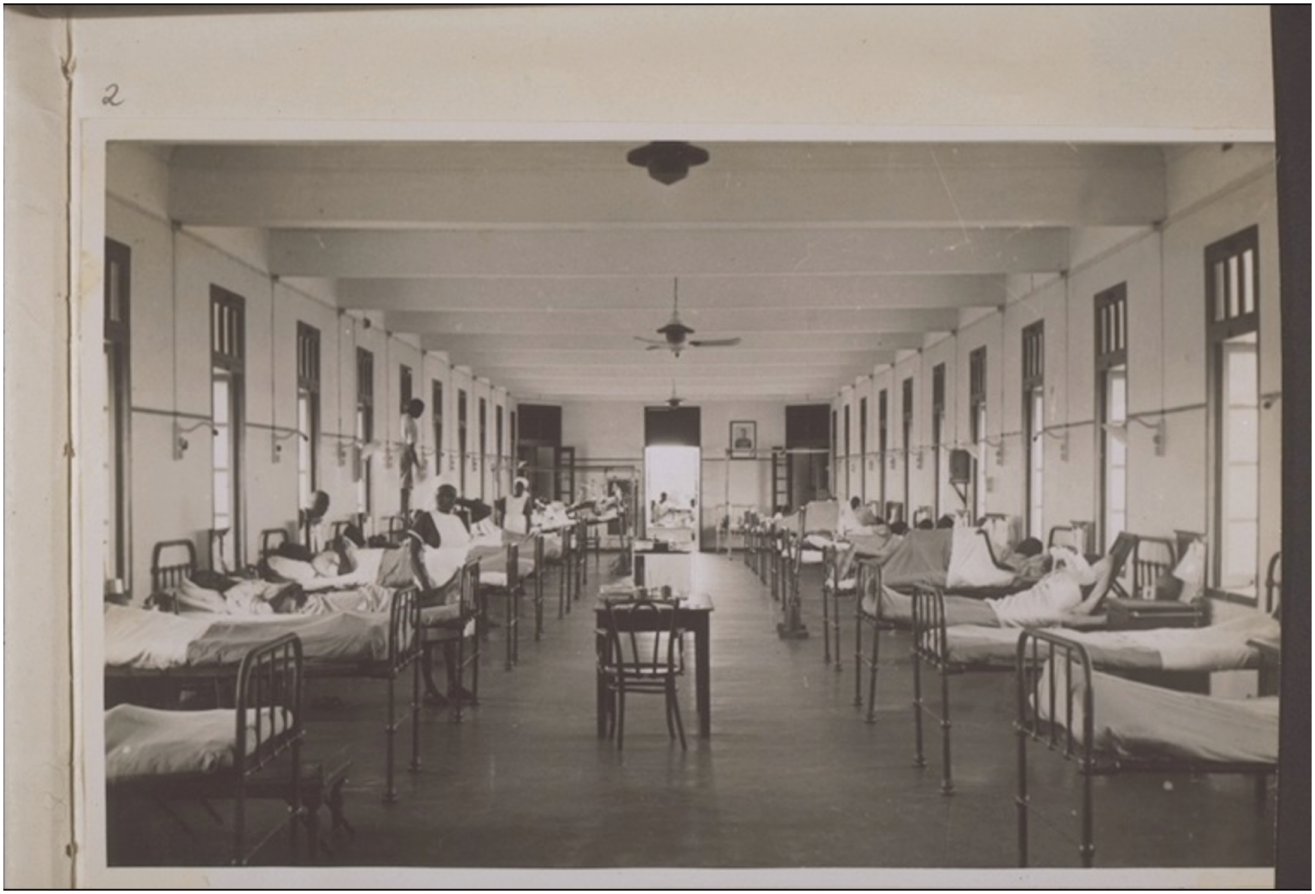
A ward at Korle Bu Hospital, c. 1940s (West African Photographic Service, Accra). Courtesy of Basel Missionary Archive, BMA QD-30.102.0002. See also Roberts p. 211.

Opened in 1923, with nurses and dispensers trained here from the start, Korle Bu was considered ‘the finest hospital in West Africa’.^40^ ‘Situated well out of the town but easily accessible’, as one Medical Officer put it, Korle Bu was removed from the old town by the Korle Lagoon.^41^ Like the university campus at Legon, the hospital complex was both spatially and architecturally distinct from the communities which it was built to serve (Figure 2). Reminiscences from early employees—a rare source of experiential data, compiled in a 1973 pamphlet and released in celebration of the hospital’s jubilee—emphasised that Korle Bu was an entirely new type of therapeutic space. Jonathan Roberts’ close reading of this unique publication has illustrated how the material environment shaped both doctors’ and patients’ experience of biomedicine during these early years.^42^ As Roberts explains, Korle Bu was designed according to the latest scientific principles, learning here was an inculcation into modernist biomedical doctrine. Patients passed through an ablution block, where they were bathed and disinfected by nurses prior to admission; staff had to navigate other, totally aseptic spaces, such as surgery theatres, which required washing and changing in anterooms. Open-plan ‘Nightingale’ wards facilitated the surveillance, stratification and segregation of the sick (Figure 3). Staff reminiscences emphasise the affective distinction of the hospital as a unique, high-technology space—an electric generator allowed for an interior lift, laundry machines, x-rays and iron lungs, and meant that, unlike elsewhere in Accra, the lights at Korle Bu were always on.^43^ As with hospitals everywhere—although perhaps especially true of spaces where biomedical interventions were relatively new—these could be intimidating spaces. For Ghanaian doctors, learning and practicing medicine here also meant learning to mediate this context. One physician, F.V. Nanka-Bruce, recalled that

when I returned from Edinburgh as a qualified doctor it took us many hours, and even days, to beg and coax a would-be patient to submit to an operation for his own benefit. In fact, we spent more time coaxing the patient than in performing the operation.^44^

When it was built, Korle Bu materialised new therapeutic ideals and promoted efficiencies made possible by modern technology and imperial integration. Generations of dispensers, nurses and physicians have since learned their craft in—and in conjunction with—buildings which were not simply symbols of biomedical advancement, but which actively ‘anticipated and produced medical practices’.^45^

The later consolidation of medical education was often similarly reliant on imperial infrastructure. KNUST has its origins in late-colonial plans for a technical school in Kumasi.^46^ With construction beginning in the late 1940s, the oldest parts of the KNUST campus are standout examples of modernist ‘tropical’ architecture.^47^ Although inspired by the dawning of African independence, and an important break from the more formal and overtly ‘European’ style employed in the construction of earlier institutions, including at Legon and Korle Bu, the modernist turn was still bound up in an imperialist discourse which emphasised the disciplinary potential of the built environment. Jane Drew, the pre-eminent British modernist working in West Africa, whose commissions included several elite secondary schools in Ghana and the University College Ibadan, the Nigerian correlate of Legon, designed buildings to be ‘deliberately geometric and orderly because such a statement is required against the extreme disorder of the luxuriant forest’.^48^ Similarly, the KNUST campus was described by its first principle as lying in a ‘tract of tropical forest … originally hidden in dense jungle’.^49^ James Cubitt and Partners, the British firm responsible for most of the original buildings, took inspiration from Kumasi’s landscaped ‘European quarter’, going on to explain that ‘landscaping was one of the first things we considered [at KNUST], perhaps by this confessing ourselves very English’.^50^ Perhaps unsurprisingly, some students remarked on the alienating effects of such spaces. In a semi-fictitious memoir of his time as a student at Ibadan, the Nigerian novelist Wole Soyinka contrasted the ‘scrupulously geometric’ new campus with fonder memories of the ramshackle, repurposed huts which made up the temporary campus, primarily because of their ‘openness to the real world’.^51^

Although the development of medical faculties emerged in historical moments removed from the context of colonisation, the cloistered, colonial-era spaces of Legon and Korle Bu have remained the epistemic centre of Ghanaian medicine. With UGMS already providing a British model of academic medical education, the later establishment of faculties in Kumasi and Tamale were intended to complement the education on offer in Accra. In practical terms—and in something of a parallel to the way in which colonial medical education was designed to be subordinate to that received in British universities—KNUST and UDS were designed to offer ancillary, epistemically subordinate forms of education in more peripheral geographies.^52^ For instance, medical training at KNUST began in the 1970s, during the Supreme Military Council’s ostensibly Marxist military dictatorship. However, and in line with its colonial foundations as a technical university, KNUST’s remit focused on applied science, rather than the more fundamental, academic education provided in Accra.^53^

Opened in the late 1990s, in the historically poor and peripheral northern savannah, the medical faculty at UDS was likewise designed to produce adequate numbers of trained health personnel with appropriate intellectual values and beliefs who, by staying and working in the rural areas, will help find solutions to the predominant and peculiar medical problems of the deprived areas.^54^

This was a period in which the IMF-led Structural Adjustment of the Ghanaian economy had significantly curtailed state expenditure and demanded a return to multi-party democracy. The founding of UDS during these years was, at least to some extent, also a means to bolster Northern support for Jerry John Rawlings’ incumbent NDC government in the return to elections. However, and as was also the case in Accra 30 years prior, financial constraints led to a necessary reliance on colonial-era infrastructure, with UDS’ first medical students using classrooms at the old School of Hygiene and sleeping in dormitories at Nyangpala, an old agricultural college. As in Accra, this reproduced a similar social removal from the city. Busses to Nyangpala stopped running in the afternoon, but if there was a party in town some students would still elect to walk the 15 km home. As also in Accra, these spaces of education were built to corral students in shared experience and to instil practices, ideas and ideals which are, necessarily, derived from the context of their construction.

In what is an important affective break from these older infrastructures, today UDS’ medical school is spread across an entirely new campus at the edge of Tamale, with clinical training taking place in the city’s recently modernised teaching hospital.^55^ In fact, both UDS and KNUST have largely outstripped their original purpose, providing relative educational parity with UGMS, and housing scholars whose research challenges the primacy of Legon and of universities in the old imperial metropoles. Yet the allure of more historied institutions still plays some role in student’ imaginations. ‘Legon, Tech, or death’—a well-known phrase amongst graduating high-schoolers anxiously awaiting their university placements—is evidence of precisely this.^56^ In some respects, and despite the development of world-class faculties elsewhere in the country, UGMS’s cloistered, colonial-era spaces have remained the epistemic centre of medical education in contemporary Ghana. Scientific standardisation and epistemic subordination were built into these infrastructures from the start; written by the colonial administration in support of the campus at Legon, one 1946 report explained that ‘a university is a great national institution, intimately affecting, and effected by, the ideals of the Government’.^57^ That many future generations of UGMS students will train in the new, flagship teaching hospital which has recently opened on the campus at Legon speaks to the enduring influence of colonial-era state building, and the epistemic centralisation which it was intended to support.

## Books

In a 1965 parliamentary debate on the legalisation of cadaveric dissection at UGMS, J.A. Bramiah, MP for Gonja East, exclaimed ‘how wonderful it will be if people of other races and other countries will come to Ghana for the treatment of their diseases, especially tropical diseases, by our doctors trained in our medical school in its African setting!’^58^ Despite such enthusiasm, epistemic authority over diseases which are endemic in Ghana but largely absent from the Global North has continued to derive primarily from historic, imperial centres of science. Published some 60 years after this parliamentary debate, all of the suggested readings in the section on tropical and infectious disease in the 2016 UDS medical student handbook were published in either Europe or North America.^59^ Although their original conclusions have been softened over the course of several editions, many of these texts reproduce the authority of colonial-era physicians; by virtue of this history, most reproduce the construction of ‘tropical medicine’ as spatially removed and ontologically distinct from medical practice in the ‘temperate’ world. Although the ideas reproduced in these materials is often the result of African scientific endeavour, textbook considerations of tropical medicine rarely reflect this and are, instead, the endpoint of a process by which knowledge of health concerns specific to Africa—as well as other postcolonial spaces—has been collated, classified, codified and otherwise subsumed by epistemic centralisation in academic publishing, if not also by the abstract universalism which lies at the heart of biomedical epistemology. This is a process which has always denied accommodate epistemologies which may bear relevance to caregiving but which exist outside of these frameworks. In the Ghanaian context, for instance, these may include traditional approaches to healing and herbalism, or indigenous codifications, nomenclatures and aetiologies of disease. Instead, the continuing and often entirely practical prescription of foreign textbooks reinforces Europe and North America as the rhetorical centres of knowledge regarding African health.

Even in forwarding some geographic aetiology, ‘tropical medicine’ naturalises the persistence of diseases which have often only endured because of structural disadvantage and historical exploitation. Many textbook examples of tropical disease (tuberculosis, malaria, leprosy, measles) were only eradicated from Europe in the nineteenth century, and only in response to broad improvements in living standards, public health interventions and the expansion of curative medicine. As Nancy Leys Stepan has argued, epidemiological shifts in Europe dovetailed with the colonisation of the tropics, as well as the advent of technologies which allowed for the reproduction of images that sensationalised diseases only recently forgotten by metropolitan physicians.^60^ For instance, one somewhat surprising inclusion in the UDS handbook is the 1913 edition of Castellani and Chalmers’s *Manual of Tropical Medicine*.^61^ This textbook clearly illustrates the early development of tropical medicine as a means to ameliorate European illness in the ‘white man’s grave’, rather than to provide aid to the colonised. Indeed, Chalmers’s introduction to the application of medicine in West Africa came during his tour as a Medical Officer, fighting for the British against the Asante during the 1900 War of the Golden Stool.^62^ In their section on malaria, Castellani and Chalmers conclude that ‘native races’ were ‘partially immune hosts [who] act as reservoirs or carriers’, enabling ‘the parasite to complete its life-cycle without producing marked pathological changes in the host’. This, as Warwick Anderson has explained, fashioned ‘native races as biological reservoirs to contain local disease organisms’.^63^ By this logic, tropical disease derived as much from the environment as from the people that live there. With the formal extension of empire from late nineteenth century, popular discourse emphasised the ‘diseased heart of Africa’ as a justification for European government, with the spread of biomedicine as part of this universal salve.^64^

Although never entirely successful, the subsequent extension of biomedicine was always, at least in part, also an attempt at epistemic erasure. The socio-spiritual basis of health common across West African intellectual traditions was often at odds with the individualism of early twentieth-century biomedicine, with its mechanistic approach to the body and emphasis on healthful individuals forming part of a virile, obedient population.^65^ Notwithstanding important spatial deviations, nineteenth-century healing practices employed in what is now Ghana ranged from the preventive to the curative, and included bone-setting and inoculation; the ritual appeasement or invocation of ancestors and deities; and the prescription of herbal remedies, as well as amulets and other spiritual medicines.^66^ The substitution of these pre-existing practices and epistemologies was a necessary part of the imperial project. Legislation passed in the 1930s, for instance, sought to restrict the influence of African healers already adjusting to epistemic change and the introduction of new medical technologies. Ordinances relating to midwifery banned unregistered midwives from ‘habitually’ attending childbirth within the limits of the town. Later legislation explicitly banned herbalists from offering injections.^67^ Through literacy, licensure and the establishment of professional associations, traditional healers adjusted practices in response to the technocratic and bureaucratic landscape laid out by the colonial state.^68^ Rather than epistemic erasure, the ultimate effect of these various forces was the hybrid, synergistic and pluralistic health system common across contemporary Ghana.^69^ Nevertheless, during the early twentieth century the imperial government sought to concentrate healthcare provisioning around the small, but steadily expanding, cadre of biomedical practitioners trained in Europe, or under European direction. Regardless of background or experience, doctors seeking employment in the colonial medical service had to first complete a certificate course at either the London or Liverpool school of tropical medicine. Here, far away from the practical, localised enactments of illness, tropical disease was enacted in theory, in the lecture theatre and in textbooks. Today, as throughout the twentieth century, Ghanaian educators recommend *Manson’s Tropical Diseases* to their students. Now in its 23rd edition, and although heavily revised since its first publication in 1898, its endurance illustrates the obduracy of tropical alterity within medicine, as well as the enduring influence of Patrick Manson, the founder of London School of Hygiene and Tropical Medicine.^70^

In recent years, historians of science have emphasised the role of African intellectual tradition in the production of the biomedical sciences. This work challenges the earlier, broadly ‘postcolonial’ construction of tropical medicine as a coherent, monolithic form of ‘imperial science’ working for imperialist ends.^71^ While such revisionism is entirely necessary with regard the *production* of tropical medicine, its *reproduction*—the subsequent codification, classification and dissemination of consensus—has often involved a much greater degree of metropolitan direction. In this respect, the material reproduction of medicine often actively worked to strip scientific advancement of any non-European influence. The International Classification of Diseases (ICD), for instance, has sought the standardisation of pathology since the end of the nineteenth century.^72^ Imbibing these textbook taxonomies is a transformative element of biomedical education, amounting ‘almost to learning a new language’, something which justified the inclusion of Latin in the curricula of elite colonial-era schools.^73^ Yet, as Clapperton Mavhunga has, for instance, shown in the history of *gopé* (trypanosomiasis, or sleeping sickness) in Zimbabwe, the Latinisation of the both the disease and its vector, the *mhesvi* (tsetse fly or, in Latin, one of a number of the genus *glossina*), worked as a ‘technology of erasure, completely covering the tracks of knowledge so that its source could never be known, never be traced’.^74^

At the same time as African knowledge of disease was recentred around metropolitan networks of science and technology, African therapeutics were subject to the same process. Although local knowledge of healing plants was readily recognised by colonial Medical Officers, chemical analyses and drug development was expected to take place in Britain.^75^ Cures, when they were produced, were codified under metropolitan direction. Dispensers training at Korle Bu in the 1920s, for instance, were given William Whitla’s *Elements of Pharmacy, Materia Medica, and Therapeutics.*^76^ Working to standards set out in British Pharmacopeia, Whitla only provided information on the drugs available within the British market; between editions, individual compounds were added or removed according to metropolitan direction.^77^ Western authority over African therapeutics did not end alongside empire. In one telling example, offered in Abena Osseo-Asare’s recent exploration of this history, Ghanaian scientists hoping to control the development of c*ryptolepis sanguinolenta* as an antimalarial drug found that the pull of more stable networks and more advanced technologies was enough to reorient their research around Western laboratories.^78^

These issues have long been acknowledged by the African academy, and there have been repeated efforts to resist historic networks of epistemic authority. Often these have concentrated on the production of texts used to disseminate ideas. The *Ghana Medical Journal* began publication in 1962, several textbooks have been written by Ghanaian educators and, latterly, several African-oriented journals of medical education have also been established.^79^ Today, as ever, students and faculty contribute to, discuss and distribute research conducted in Ghana and by Ghanaian faculty—from Fred Sai’s world-leading public health research from the first years of UGMS, through to Juventus Ziem’s research into oesophagostomiasis, a parasite localised to the north Ghana and Togo, and hosted by UDS in the 2000s.

However, as the UDS handbook suggests, these efforts have struggled to significantly alter the material episteme of tropical medicine. This is, in no small part, due to the long-standing economic constraints which have defined the development of medical education in postcolonial Ghana, and which continue to constrain national and institutional capacity to exercise epistemic autonomy in all fields. In pursuit of ‘scientific and technical equity’ with the West, in the 1960s Nkrumah had intended for UGMS to be ‘planned, directed and operated by Ghanaians without any external assistance in men or money’.^80^ Yet, shortly after this, the nucleus of the UGMS medical library was created out of donations from the British government, with further donations drawn primarily from universities and charities in Western Europe and the USA.^81^ Later, during a 1987 conference in Brazzaville, regarding ‘African Perspective on Medical Education’, attendants universally stressed the need for locally made textbooks and teaching tools.^82^ However, this was a period of severe economic and political crisis in Ghana, foreign exchange was extremely limited and university budgets were easily exhausted on expensive, imported equipment. UGMS’ was quickly spent on anatomy models produced in the UK, replicating machines from Japan and laboratory equipment made in the USA.^83^ By this time, the medical library—largely built from the donations received 20 years earlier—was in a ‘deplorable state’ and required a new appeal for donations from the UK’s Overseas Development Agency.^84^ This cyclical process, donation necessitated by decay, is part of an entrenched history of material-epistemic dependency in biomedicine. Even into the 2000s, much of the UDS medical faculty has been made material via donations from Nuffic, a Dutch NGO which works towards ‘internationalisation in education’.^85^

As the most immutable of mobiles, textbooks depend on the authority of stable networks and powerful actors—on eminent professors, feted universities and wealthy publishing houses. The stability of these networks and the power of these actors is necessarily historical and, with respect to tropical medicine in particular, is necessarily bound to a history of epistemic subordination and imperialist othering. A significant amount of labour is required to resist these historied materials and, in spaces which are often defined by shortages of time, money or both, material attempts to resist the gravity of the epistemic centre are often the most fragile. During the 1980s economic crisis, for instance, the *Ghana Medical Journal* was, for several years, forced to cease publication. Today at UDS, as in many universities around the world, students will commonly refer to ‘soft-copies’ of the textbooks which are listed in their course manuals. The prohibitive cost of medical textbooks means that pirated PDFs are popular, shared via USB drives or WhatsApp groups. Photocopying is also a lively business on and around university campuses across Ghana, and one with a longer history. Although these forms of material-epistemic reproduction work to resist the marketisation of scientific knowledge, they nevertheless also reproduce the epistemic authority of historic and often imperial centres of science. As has been shown in several studies of African science, material shortcomings, and the related loss of capacity leads actors to site ‘real’ science somewhere else, in an earlier time or a different place.^86^ The ‘material absence’ or, at least, the material subordination of digital, photocopied or tattered textbooks simply reaffirms the ‘abstract presence’ of an imagined centre of biomedical practice, something which is often also woven into the science which is reproduced between their covers.^87^

## Bodies

In 1980, in the midst of Ghana’s economic crisis, a team of professors and administrators from UGMS noted that ‘although Komfo Anokye [Teaching] Hospital [KATH] abounds in excellent clinical material, i.e. patients with diverse diseases most suitable for teaching purposes, the conditions prevailing at present in the Hospital are not conducive to the efficient clinical instruction of medical students’.^88^ Heavy inflation and the outmigration of hundreds of qualified doctors in previous years had led to a severe shortage of both staff and equipment at KATH—the teaching hospital affiliated with KNUST—as well as to questions over its ability to provide adequate medical education. Only a few years after the medical faculty’s establishment, these shortages had led, in turn, to student complaints ‘that there is a lack of emphasis on practical training, but undue emphasis on theory’.^89^ Furnishing medical students with the requisite number and variety of patient bodies continues to prove an acute logistical challenge. Today, in Tamale Teaching Hospital, where UDS students complete their rotations, 400 students occupy a 600-bed hospital, with only 20–30 specialist physicians on staff. Here students on clinical rotation often note that, although they have observed interventions or attended ward rounds where knowledge of patient health was largely derived from some physical interaction with the patient’s body, they had not necessarily been invited to listen to or feel for those same things. This final section takes that there are certain materials which can be readily employed to localise biomedical epistemology: the knowing, skilled bodies of experienced physicians; the known bodies of their patients; and the technologies which are needed to observe, preserve and reproduce local biologies. However, in settings where student access to clinical material is restrained by shortage or by pedagogy, students are introduced to the standardised and universalised bodies which are described in textbooks, depicted in audio-visual resources, and which are made material in the simulated and model bodies which are a growing presence in medical schools around the world. As we have already seen in the buildings and textbooks used in the transmission of knowledge, these materials are not impartial, but are agential objects, scripted by their own material histories and by necessarily historical ideas regarding their use and interpretation.

Shortages of clinical material in medical education is a relatively modern phenomenon in Africa, as everywhere, and reflects increased student enrolment as well as shifting ethical concerns regarding the use of post-vital material and student access to patients.^90^ When UGMS was established in the 1960s, however, its first students were readily furnished with the materials considered necessary for an academically oriented education along the lines of a British medical school. This included ready access to cadavers and the conservation of preserved specimens, some of which were housed in the W.N. Laing Pathology Museum. The 1965 Anatomy Act quickly swiftly through parliament so the new school could obtain unclaimed or donated bodies for dissection. In the early years of UGMS, there were only around five students to a cadaver.^91^

**Fig. 4.**
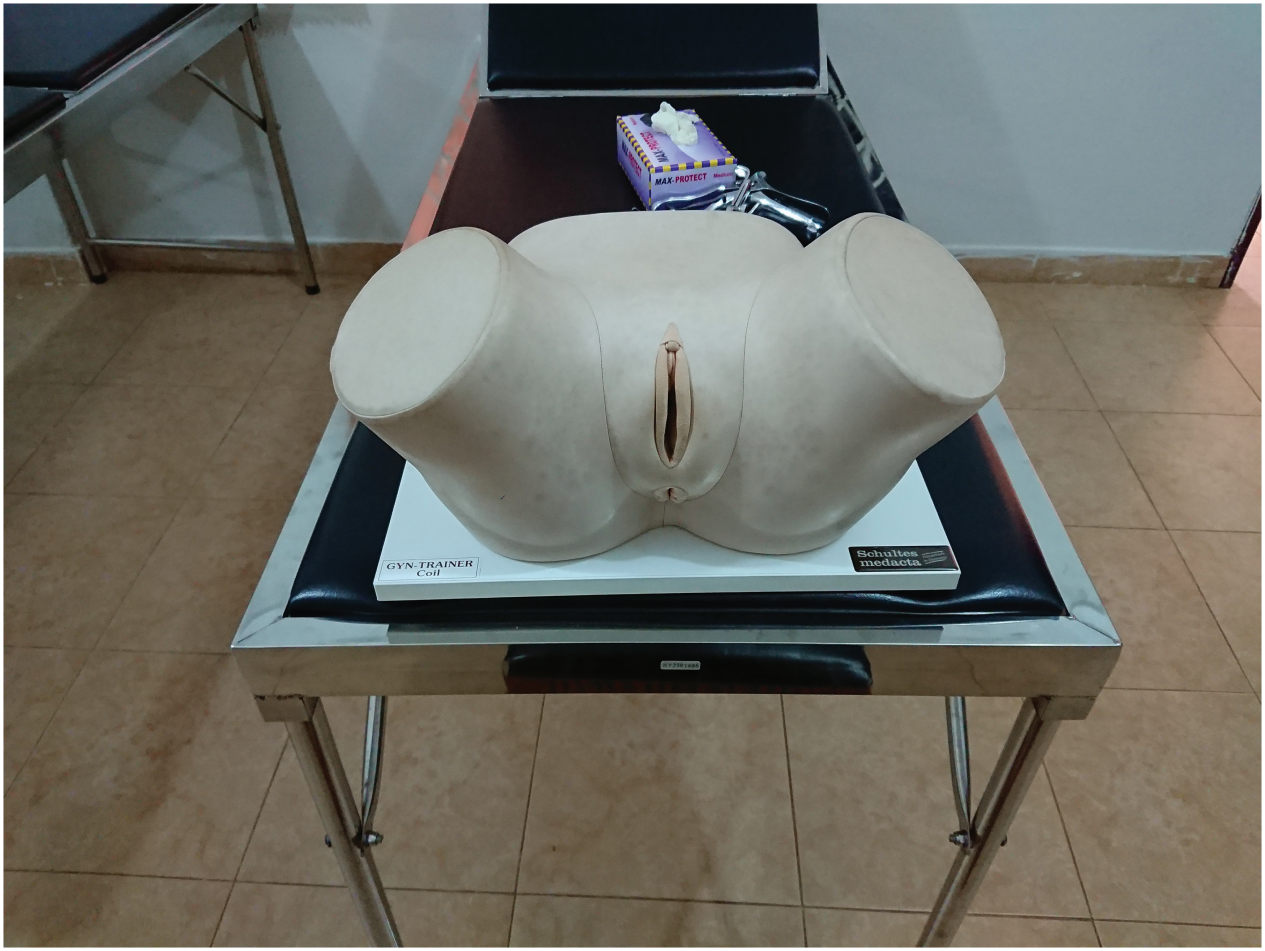
Schultes Medacta gynaecology manikin set up for a Skillslab class at UDS in 2018 Andrea Wojcik.

**Fig. 5.**
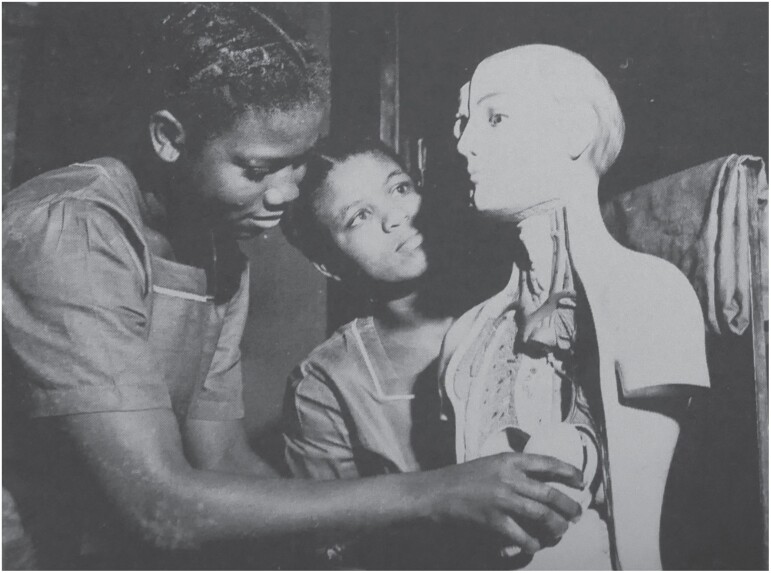
Nursing students, UGMS, c. 1970, from Korle Bu Hospital 1923–73: Golden Jubilee Souvenir (Accra: Korle Bu Hospital Celebration Committee, 1973).

In Tamale, by the late 1990s, UDS’ access to dissection material was much more sporadic.^92^ In the intervening decades, educationalists everywhere had also begun to question the necessity of dissection in medical education. Instead of pursing the more traditional medical curricula found at UGMS or KNUST, in 2007 UDS adopted a programme of Problem-Based Learning (PBL) and constructed as ‘skills laboratory’, or Skillslab, for students to practice clinical skills and to learn through the ‘problems’ which they are likely to face in practice.^93^ As a result, and unlike in Ghana’s older schools, patient simulations became a central part of UDS’ pedagogy. From their very first year, students learn the theoretical tenets of medicine while also practicing physical skills. Students practice on themselves or each other; occasionally on trained ‘standardised patients’, volunteers or employees enrolled to guide student examinations; and often on the rubber and plastic dolls which are used to safely and ethically imitate breast, prostate, gynaecological or obstetric exams. These models are imported, primarily from European manufactures and, as such, tend to reproduce a European patient public. At the most obvious, aesthetic level, this often means that students’ model ‘patients’ have light skin. Although Schultes Medacta, the German manufacturer of most of UDS’ trainers, do now produce manikins with darker skin, older faculty recall that the teaching manikins used across Ghana had always been white, and some manikins still are (see Figures 4 and 5). Similarly, illustrations of light-skinned bodies predominate in the textbooks commonly used across all three faculties. As one recent study has shown, light-skinned bodies are overrepresented—even in relation to the USA’ demographic make-up—in as many as three quarters of the most assigned medical textbooks.^94^ Unsurprisingly, students and teachers at UDS often note the challenge of finding representative photographic material of dermatological conditions or symptoms of systemic disease as they appear on darker skin, either in textbooks or via online image searches.

A less visible issue is the reproduction of spatially and historically specific biologies in the design of such manikins.^95^ Take, for instance, the Schultes Medacta gynaecology trainers used at UDS (Figure 4). Cut off at the thigh and midriff, these manikins limit ‘patients’ to their reproductive organs. Each represents a different woman: a young woman yet to carry a child, a woman with several children already, an expectant mother, an older woman and a woman attending the clinic at an opportune time for the insertion of an intrauterine contraceptive device (IUD). Meet ‘Nullipara’, ‘Multipara’, ‘Gravida’, ‘Seniora’ and ‘Coil’. Each trainer has a crib sheet which details what a student should be feeling as they conduct their examinations. Each has specific anatomies and, in some cases, pathologies which students learn to recognise. These models are intended to offer both sensorial training and training in the performance of gynaecology.^96^ Students, for instance, learn to feel for healed scars, fibroids and cancers. Although disease is not universal in either incidence or social construction, models produced in Europe encourage students to sense pathologies, like uterine cancers, which are often significantly less common in Ghana than in Europe.^97^ Perhaps more illustrative of this point is the ‘Coil’ model, in which we can see the messy politics and past lives of contraceptive technologies in African history.^98^ IUDs tend to be developed and marketed by Western pharmaceutical firms and, in Africa at least, are often promoted by international NGOs concerned with family planning, if not population control.^99^ As with gynaecology textbooks, which have been found to commonly downplay health risks associated with IUDs, such manikins shape medical pedagogy through the tacit approval and normalisation of pharma-contraceptives.^100^ In both examples, the UDS manikins speak to the spaces and times in which they were developed, as well as to unproven assumptions of universality in demographic and epidemiological change.^101^ When technologies encourage students to feel for disease, or for the optimum conditions for applying (or recommending) contraception, students also feel the histories, philosophies and subjectivities which have gone into their construction.

As was also the case with written material, providing students with access to clinical matter and to bodily materialisations local to Ghana has often come with significantly higher costs in terms of both time and money. For instance, and as mentioned in the introduction, the W.N. Laing Pathology Museum closed in the early part of the twenty-first century. Its space was first encroached upon by the construction of a lecture theatre, equipped with heavy curtains to facilitate the use of audio-visual aids, most of which had been donated by British universities.^102^ The time needed to prepare and preserve specimens, the cost of the necessary chemicals and their ready black market in Ghana (where funeral rites might entail that bodies may wait some weeks before they are interred) has meant that even maintaining the museum’s collection has been prohibitively costly. Where students had once studied specimens alone, or in a small tutor groups, today’s students learn from depictions of pathologies which are found online or in textbooks. As with the use of cadavers or cell slides, the pathology museum was intimately local and built on relationships with the local community, even if the broader technoscientific assemblage—both material and conceptual—was not. As in pathology education, the use of cadavers in anatomy classes and the local production of histology slides has also been eroded by the growing availability and cost-effectiveness of audio-visual and digital teaching tools and methodologies. The ascendency of audio-visual and then digital materials have, more often than not, worked to hasten the decay of the local in favour of an abstracted universal body which is typically drawn from European or North American contexts.^103^

A similar story can be found at UDS. In the space between faculty buildings at UDS lies an empty shipping container which had once been set up with a video link and projection facilities in order to allow students to watch surgeries in the regional hospitals that they would soon be staffing. Today it tells a similar story of decay. Unreliable internet connections on both sides of the line led to the eventual abandonment of a novel attempt to localise training. As with the pathology museum at UGMS, or the *Ghana Medical Journal*, material solutions to the localisation of medicine are persistently undermined by their expense—in terms of time, money and expertise—as well by the gravity of historic centres of medical epidemiology. Yet UDS has been successful in its remit to keep doctors in northern hospitals, and to instil biomedicine in a space which had always been peripheral within its epistemic framework. These successes, it seems, are not drawn from the material environment of the medical school but from innovative, practical efforts to introduce students to local enactments of medicine. Since 2007, UDS has instigated a programme of Community-Based Education and Service (COBES), in which students spend 4 weeks of every year in primary health centres or district hospitals in various rural communities. The effect, it seems, is increased interest in the work of rural doctoring across the north.^104^ Locating medical education away from the medical school offers space for sincere interactions with localised presentations of disease as well as well as local understandings of health. It is, perhaps, the escape from the material surroundings of the medical school—and their affective, material histories—which provides the necessary epistemic break.

## Conclusion

Writing in 1969, Charles Odamtten Easmon, the first Dean of UGMS, explained to the readership of the *Ghana Medical Journal* that, ‘during the past five years, we have made many mistakes … the outstanding mistake was the adoption of a modified traditional British curriculum’. This curriculum had, in Easmon’s opinion, mired the development of an alternative programme of instruction, one which was ‘revolutionary in concept and African in execution and orientation’.^105^ For Easmon, a revolutionary African medical school would move away from didactic, lecture-based education, in favour of student-led inquiry sited in the varied clinical contexts found throughout Ghana. The immense labour which had gone into the foundation of a school which operated independent of the former colonial administration was apparently not enough, a more fundamental reorientation of medical epistemology was also required. Educators recognised that any such reorientation would also require greater agency over the material conditions of science. Not far away from Ghana, Marc Sankale, the director of instruction at Senegal’s *Ecole de Médecine*, argued that African medicine would only be independent if it were freed from technical dependence on international donors. Embodying what has been described as a new, nationalist form of technopolitics, Sankale explained that

we must know how to ask: what economic interest, what political calculus, what new technique of espionage hides itself behind each radiological apparatus. If they offer us an operating table, we should ask ourselves what genre of amputation it is intended to serve!^106^

The nationalisation of science education, as well as the materials necessary for scientific discourse, was part of this broader effort. As Nkrumah wrote in its inaugural, 1962 issue, ‘the *Ghana Medical Journal …* will afford the means of disseminating medical knowledge among yourselves and your colleagues through Africa and beyond’.^107^ Notwithstanding this early support—as well as the remarkable, ongoing expansion of national capacity to train doctors—the material culture of medical education has never been ‘revolutionary in concept’, nor ‘African in execution and orientation’. Indeed, this material culture should perhaps be seen as agential force which agitates against any such radical change.

In Ghana, the ever-growing number of practicing physicians and researchers have worked to recentre medical care and the *production* of science in intimately local contexts and in response to intimately local needs. However, as we have seen, the buildings, books and bodily representations which *reproduce* biomedical epistemology have rarely reflected the same situatedness of health and disease, nor the fluidity of biomedicine in practice. Indeed, by imploding the material culture of medical education in modern Ghana, we see the outdated but intractable ideas which endure in the material conditions of leaning, and which contribute to affective understandings of science in the present. For instance, and despite the massive expansion of educational infrastructure following Ghanaian independence in 1957, it is the colonial-era buildings at UGMS, Korle Bu and KNUST which have persisted as the epistemic centre of Ghanaian medicine. Even in newer universities, like UDS, which were established long after the end of empire, schools and their students have remained reliant on Western publishers for their textbooks and on Western manufacturers for their anatomical models, audio-visual materials and for most of their other educative technologies. This tends to be the case even when such materials trade in the elucidation of those ‘tropical’ diseases usually unseen in Western hospitals. The alien origins of these materials means that the sort of bodies reproduced in, and the practices encouraged by these teaching tools rarely represent the patients and practices which Ghanaian students will come to encounter once they graduate. Greater access to patient bodies and clinical practice undoubtedly works to resist the epistemic standardisation promoted by imported materials, as well as the epistemic gravity of Europe and North America. However, in settings where student access to clinical material is restrained by shortage or by pedagogy—as is currently the case all around the world—the Western origin and orientation of teaching tools contributes to a distracting epistemic imaginary which reinforces the historical peripherality of medicine outside of old, often imperial centres of science.

Walter Mignolo, one of the most prominent recent advocates for the decolonisation of knowledge, has forwarded ‘epistemic disobedience’ as a means to disrupt the authority of the European intellectual tradition.^108^ Disruption of the material assemblages of scientific tradition has, however, rarely been considered part of this project. This is perhaps because, within the social study of science at least, focus on immutability and material dependency in colonial and postcolonial contexts is often taken to deny the agency of local actors. By contrast, attention to the fluidity of science and technology recognises ‘the appropriations, resistances, transformations and contestations occurring as science and technology travel over the uneven terrain that colonialism has worked’.^109^ While such considerations are clearly integral to a more accurate, global accounting of the history of science, they are also apt to downplay the technologies which work to restrict the agency of those at the geopolitical periphery, or at the periphery of the actor-network. These are technologies which work to standardise science, its history and epistemology, and to even the terrain that colonialism has worked.^110^

In between literatures which emphasise either material fluidity or epistemic dependency, there is room for finer distinction in the study of material constraints on human agency. Where and why constraints occur offers valuable insight into the nature of specific technologies and the regimes in which they operate. Sited at one end of this spectrum, the relative immutability of educational materials actively cultivates a degree of epistemic obedience. While there is a degree of labour involved in the effective translation of these technologies, considerably more time and money would be required to resist them entirely. Where they have occurred, conscious efforts to localise teaching materials have tended to rest on relatively fragile actor-networks, are difficult to scale up, and are often the first to erode. The closure of spaces like the W.N. Laing Pathology Museum also contributes to the erasure of Ghanaian histories of science, and to the loss of material cultures which defy the placement of African science at the edge of an epistemic empire. Future attempts to decolonise the university might, then, also give greater consideration to the material culture of education, to the proselytising nature of teaching materials, and the various technologies used in the reproduction of science.

## Footnotes

1 For more details on the history and current standing of the museum, see Robert Kumoji and John Nott, ‘The Pathology Museum and Korle Bu’, in John Nott and Anna Harris, eds, *Making Sense of Medicine: Material Culture and the Reproduction of Medical Knowledge* (Bristol: Intellect, 2022), 281–94.

2 Today, the application of advanced methods in diagnostic pathology at UGMS—such as the immunostaining of specific proteins or the isolation of genetic influences—continues to be hampered by the cost and availability of reagents in Ghana. See J. T. Anim, ‘The Pathologist in Ghana and Potential for Research’, *Ghana Medical Journal*, 2018, 52, 103–11; elsewhere, in Britain for instance, only a handful of the eighty-plus medical museums open in 1950 are still operating. See Elizabeth Hallam, *The Anatomy Museum: Death and the Body Displayed* (London: Reaktion Books, 2008).

3 John Iliffe, *East African Doctors: A History of the Modern Profession* (Cambridge: Cambridge University Press, 2002); Adell Patton, *Physicians, Colonial Racism, and Diaspora in West Africa* (Gainesville: University Press of Florida, 1996); Adelola Adeloye, ed, *Nigerian Pioneers of Modern Medicine: Selected Writings* (Ibadan: Ibadan University Press, 1977). For the general history of biomedicine in Ghana, see K. David Patterson, *Health in Colonial Ghana: Disease, Medicine, and Socio-Economic Change, 1900–1955* (Waltham, MA: Crossroads Press, 1981); Stephen Addae, *The Evolution of Modern Medicine in a Developing Country: Ghana 1880–1960* (Bishop Auckland: Durham Academic Press, 1996).

4 Claire L. Wendland, *A Heart for the Work: Journeys through an African Medical School* (Chicago: The University of Chicago Press, 2010), 27.

5 Wendland, 197–200.

6 Wendland, 157–60.

7 Delanyo Dovlo and Frank Nyonator, ‘Migration by Graduates of the University of Ghana Medical School: A Preliminary Rapid Appraisal’, *Human Resources for Health Development Journal*, 1999, 3, 40–51.

8 S. Eliason and others, ‘Migration Intentions of Ghanaian Medical Students: The Influence of Existing Funding Mechanisms of Medical Education (“The Fee Factor”)’, *Ghana Medical Journal*, 2014, 48, 78–84.

9 For the author's further work to this end, see John Nott and Anna Harris, ‘Sticky Models: History as Friction in Obstetric Education’, *Medicine Anthropology Theory*, 2020, 7, 44–65; John Nott and Anna Harris, ‘Teaching the Normal and the Pathological: Educational Technologies and the Material Reproduction of Medicine’, *Science as Culture*, 2023, 32, 214–39; John Nott, ‘Architecture for Anatomy: History, Affect, and the Material Reproduction of the Body in Two Medical School Buildings’, *Body & Society*, 2023, 29, 99–129.

10 On the agency of historical materialities, see, for instance, Lorraine Daston, ‘Introduction: The Coming into Being of Scientific Objects’, in Lorraine Daston, ed, *Biographies of Scientific Objects* (Chicago: University of Chicago Press, 2000), 1–14; Karen Barad, *Meeting the Universe Halfway: Quantum Physics and the Entanglement of Matter and Meaning* (Durham, NC: Duke University Press, 2007); Pamela H. Smith, Amy R. W. Meyers and Harold J. Cook, eds, *Ways of Making and Knowing: The Material Culture of Empirical Knowledge* (Ann Arbor: The University of Michigan Press, 2014).

11 Thomas S. Kuhn, *The Structure of Scientific Revolutions* (Chicago: University of Chicago Press, 1962), 136.

12 Ann Laura Stoler, ‘Imperial Debris: Reflections on Ruins and Ruination’, *Cultural Anthropology*, 2008, 23, 191–219; Alice Street, ‘Affective Infrastructure: Hospital Landscapes of Hope and Failure’, *Space and Culture*, 2012, 15, 44–56; Sabelo J. Ndlovu-Gatsheni, ‘The Cognitive Empire, Politics of Knowledge and African Intellectual Productions: Reflections on Struggles for Epistemic Freedom and Resurgence of Decolonisation in the Twenty-First Century’, *Third World Quarterly*, 2020, 0, 1–20.

13 In this respect, this article is broadly in agreement with Simon Choat, ‘Science, Agency and Ontology: A Historical-Materialist Response to New Materialism’, *Political Studies*, 2018, 66, 1027–42.

14 Francis B. Nyamnjoh, ‘Decolonizing the University in Africa’, in Nic Cheeseman, ed, *Oxford Research Encyclopedia of Politics* (Oxford: Oxford University Press, 2019), 1–36. On the longer history of African agitation for the development of universities, see Eric Ashby, *African Universities and Western Tradition* (Cambridge, MA: Harvard University Press, 1964).

15 For an authoritative account focused on African universities more generally, see Sabelo J. Ndlovu-Gatsheni, *Empire, Global Coloniality & African Subjectivity* (New York: Berghahn Books, 2015), 177–95.

16 Walter D. Mignolo, ‘Introduction: Coloniality of Power and de-Colonial Thinking’, *Cultural Studies*, 2007, 21, 155–67 (157); Margaret M. Lock and Vinh-Kim Nguyen, *An Anthropology of Biomedicine* (Oxford: Wiley-Blackwell, 2010), 2.

17 See, for instance, Annemarie Mol, *The Body Multiple: Ontology in Medical Practice* (Durham, NC: Duke University Press, 2002); Jörg Niewöhner and Margaret Lock, ‘Situating Local Biologies: Anthropological Perspectives on Environment/Human Entanglements’, *BioSocieties*, 2018, 13, 681–97.

18 Bruno Latour, ‘Drawing Things Together’, in M. Lynch and S. Woolgar, eds, *Representation in Scientific Practice* (Cambridge, MA: MIT Press, 1990), 19–68.

19 Madeleine Akrich, ‘The De-Scription of Technical Objects’, in Bijker Wiebe E. and John Law, eds, *Shaping Technology/Building Society: Studies in Sociotechnical Change* (Cambridge, MA: MIT Press, 1992), 205–25; Nott and Harris, ‘Sticky models’, 59–60.

20 See, for instance, Ericka Johnson, ‘Simulating Medical Patients and Practices: Bodies and the Construction of Valid Medical Simulators’, *Body & Society*, 2008, 14, 105–28; Stefan Hirschauer, ‘The Manufacture of Bodies in Surgery’, *Social Studies of Science*, 1991, 21, 279–319; Rachel Prentice, *Bodies in Formation: An Ethnography of Anatomy and Surgery Education* (Durham, NC: Duke University Press, 2013); Anna Harris, ‘Listening-Touch, Affect and the Crafting of Medical Bodies through Percussion’, *Body & Society*, 2016, 22, 31–61.

21 This method is inspired by Joseph Dumit, ‘Writing the Implosion: Teaching the World One Thing at a Time’, *Cultural Anthropology*, 2014, 29, 344–62, and his method has been used in Nott and Harris, ‘Sticky models’; Nott and Harris, ‘Teaching the normal and the pathological.’ A note on the non-textual sources in use throughout, in 2019, a dozen in-depth oral history interviews were conducted with experienced medical school faculty in Accra and Tamale. In 2018, four focus group discussions were held by Andrea Wojcik and the author with medical students then enrolled at the University for Development Studies. Four months’ residence at UDS over the course of 2018 and 2019, as well as numerous visits to the University of Ghana, yielded informal conversations and observations which have also contributed to this research. All interviews, focus groups and observations were conducted on the assumption of participant anonymity but, on request, I am happy to offer more detailed information with a mind to these conditions. Andrea Wojcik’s ethnographic work on UDS is forthcoming.

22 See, for instance, Andrew Saint, *Towards a Social Architecture: The Role of School-Building in Post-War England* (New Haven: London, 1987); Roy Kozlovsky, ‘The Architecture of Educare: Motion and Emotion in Postwar Educational Spaces’, *History of Education*, 2010, 39, 695–712.

23 See Michael Guggenheim, ‘Im/Mutable Im/Mobiles: From the Socio-Materiality of Cities towards a Differential Cosmopolitics’, in Anders Blok and Ignacio Farias, eds, *Urban Cosmopolitics: Agencements, Assemblies, Atmosphere* (London: Routledge, 2016), 63–81; Anique Hommels, ‘Obduracy and Urban Sociotechnical Change: Changing Plan Hoog Catharijne’, *Urban Affairs Review*, 2000, 35, 649–76; Street.

24 R. Bagulo Bening, *A History of Education in Northern Ghana, 1907–1976* (Accra: Ghana Universities Press, 1990); R. Bagulo Bening, *University for Development Studies in the History of Higher Education in Ghana* (Accra: Centre for Savana Art and Civilisation, 2005).

25 Historians of architecture often emphasise the colonial city as a dual space, split into well-planned, quarantined areas featuring European architecture and African spaces, which were often largely ignored; see, amongst many others, Anthony D. King, *Urbanism, Colonialism, and the World-Economy* (London: Routledge, 1991); Gwendolyn Wright, *The Politics of Design in French Colonial Urbanism* (Chicago, IL: University of Chicago Press, 1991).

26 B. D. Addai, quoted in Bening, *University for Development Studies*, 94, 96.

27 Gold Coast Colony, Report of the Committee on Higher Education (Accra: Government Printer, 1946), 12–13. Quoted in Bening, *University for Development Studies*, 92.

28 Livsey, *Nigeria’s University Age*; Tim Livsey, ‘“Suitable Lodgings for Students”: Modern Space, Colonial Development and Decolonization in Nigeria’, *Urban History*, 2014, 41, 664–85.

29 PRAAD/ADM/11/1/1577, Medical and dental scholarship schemes, supervision of scholars in England, 1938–44, P. Z. Ezechiel, ‘Government Scholarships to African Students for the Purpose of Studying Medicine in the United Kingdom’, 1940.

30 PRAAD/ADM/11/1/1577, Medical and dental scholarship schemes, supervision of scholars in England, 1938–44, Letter from R. W. Stoppord, Principal, Achimota College, Accra, to Director of the Medical Services, Accra. 5th June 1942.

31 W. D. Inness, *Report of the Committee Appointed by the Secretary of State for the Colonies to Formulate a Scheme for the Establishment in British West African of a College for the Training of Medical Practitioners and the Creation and Training of an Auxiliary Service of Medical Assistants* (Accra: Government Printing Office, 1928), 6.

32 UG/1/3/2/2/129, Medical School Agenda and Minutes of Meetings, 1988–2005, Letter from R. W. Stoppord, Principal, Achimota College, Accra, to Director of the Medical Services, Accra. 5th June 1942.

33 Inness, 15.

34 Livsey, 19–40.

35 Walter Elliot, *Report of the Commission on Higher Education in West Africa* (London: HMSO, 1945), 6.

36 Nkrumah was a vocal advocate for the development of a Ghanaian medical faculty, personally writing to external funders and Ghanaian doctors working overseas to encourage their return to Ghana as teaching staff. See PRAAD/RG/11/2/64, ‘Proposals for Medical School, 1962–3’ and PRAAD/RG/17/1/152, ‘Medical Faculty, 1959’.

37 UG/1/3/2/3/15, Proposed School of Medicine, 1952–60.

38 PRAAD/RG/3/6/1095, ‘Medical school and medical centre complex, 1974’.

39 The exact reason for the abandonment of these projects is difficult to ascertain. Details of the Nkrumah-era plans can be found in PRAAD/RG/11/2/64, ‘Proposals for Medical School, 1962–3’. The school was to be funded by the US government and largely staffed by American physicians. Given Ghana’s central position in the Cold War non-aligned movement, it seems likely that this was a concern. British authorities were also unhappy with the level of US involvement. Sir Christopher Cox, and the Department of Technical Cooperation (a postcolonial forebear of the Department for International Development) were ‘not entirely happy about the adoption of the Medical School by the Americans and would like to have some voice in the development of this project’. Political and economic instability in the 1970s almost certainly necessitated the abandonment of the second plan.

40
*Korle Bu Hospital 1923–1973: Golden Jubilee Souvenir* (Accra: Celebration Committee, Korle Bu Hospital, 1973), 85.

41
*Jubilee Souvenir*, 85.

42 Jonathan Roberts, ‘Remembering Korle Bu Hospital: Biomedical Heritage and Colonial Nostalgia in the “Golden Jubilee Souvenir”’, *History in Africa*, 2011, 38, 193–226.

43 Roberts, 211–12; *Jubilee Souvenir*, 54–56, 74, 76.

44
*Jubilee Souvenir*, 70; see also Roberts, 213–14.

45 This is Annmarie Adams’ argument, made with reference to hospitals in North America. See Annmarie Adams, *Medicine by Design: The Architect and the Modern Hospital, 1893–1943* (Minneapolis: University of Minnesota Press, 2008), xx.

46 KNUST has its origins in the Watson Commission, established to investigate the cause of the 1948 riots in Accra. The development of a technical or vocational school (‘education of the hand as well as head’) was seen as a means to occupy a ‘youthful hooligan element’, since a ‘literate man so educated is susceptible to propaganda and rumour of the crudest absurdity’. Andrew Aiken Watson, *Report of the Commission of Enquiry into Disturbances in the Gold Coast, 1948* (London: HMSO, 1948), 64–65.

47 Ola Uduku, ‘Tropical Ivory Towers; a Critical Evaluation of West African Campus Planning’ (presented at the 11th Docomomo International Conference, Mexico City, 2010).

48 J. B. Drew, ‘Introduction’, *Architectural Design*, 1955, 25, 137–39; quoted in Livsey, *Nigeria’s University Age*, 74.

49 J. P. Andrews, ‘Kumasi College of Technology, Gold Coast’, *Nature*, 1953, 172, 609–10 (609).

50 ‘Recent Buildings in the Gold Coast: Architects James Cubitt and Partners’, *The Architectural Review*, 1956, 119, 230–31 (230).

51 Wole Soyinka, *Ibadan: The Penkelemes Years. A Memoir 1946–65* (London: Methuen, 1995), 177; quoted in Livsey, *Nigeria’s University Age*, 83.

52 R. B. Bening’s work is essential on this point. Bening, *University for Development Studies*; Bening, *Education*.

53 Bening, *University for Development Studies*, 28–31.

54 Quoted in Bening, *University for Development Studies*, 82.

55 On affect and medical school architecthire see, Nott, ‘Architecture for Anatomy.’

56 Here I am reminded of a line from Alan Bennett, *The History Boys* (London: Faber & Faber, 2008). When asked why, as a young man, he had tried to get a place at Oxford University, Hector, the beloved but troubled literature tutor, explains that it was because of the ‘cloisters, ancient libraries … I was confusing learning with the smell of cold stone’.

57 Gold Coast Colony, Report of the Committee on Higher Education (Accra: Government Printer, 1946), 12–13. Quoted in Bening, *University for Development Studies*, 92.

58 PRAAD/RG/11/2/82, ‘The Anatomy Act, 1965–6’, Parliamentary Debates, Second reading of the Anatomy Bill, 23rd March 1965.

59 Francis A. Abantanga, ed, *Handbook on Regulations, Programmes and Curricula for the School* (Tamale: University for Development Studies School of Medicine and Health Sciences, 2016).

60 Nancy Leys Stepan, *Picturing Tropical Nature* (London: Reaktion Books, 2001), 149–79; see also Anna Crozier, ‘Sensationalising Africa: British Medical Impressions of Sub-Saharan Africa, 1890–1939’, *The Journal of Imperial and Commonwealth History*, 2007, 35, 393–415; for an example of this forgetting, see John Nott, ‘No One May Starve in the British Empire’: Kwashiorkor, ‘Protein and the Politics of Nutrition Between Britain and Africa’, *Social History of Medicine*, 2021, 34, 553–76.

61 Aldo Castellani and Albert John Chalmers, *Manual of Tropical Medicine* (London: Baillière, Tindall and Cox, 1913).

62 ‘Dr. A. J. Chalmers’, *Nature*, 1920, 105, 271–72.

63 Castellani and Chalmers, 115; quoted in Warwick Anderson, *Colonial Pathologies: American Tropical Medicine, Race, and Hygiene in the Philippines* (Durham, NC: Duke University Press, 2006), 92.

64 See, for instance, Jean Comaroff, ‘The Diseased Heart of Africa: Medicine, Colonialism and the Black Body’, in Shirley Lindenbaum and Margaret M. Lock, eds, *Knowledge, Power, and Practice: The Anthropology of Medicine and Everyday Life* (Berkeley: University of California Press, 1993), 305–29.

65 Compare, Michel Foucault, *The History of Sexuality. Vol. 1: The Will to Knowledge* (London: Penguin, 1979) and Steven Feierman and John M. Janzen, eds, *The Social Basis of Health and Healing in Africa*, (Berkeley, CA: University of California Press, 1992).

66 See, for detail of Akan practice, Adam Mohr, ‘Missionary Medicine and Akan Therapeutics: Illness, Health and Healing in Southern Ghana’s Basel Mission, 1828–1918’, *Journal of Religion in Africa*, 2009, 39, 429–61 (437–43).

67 PRAAD/CSO/5/1/320, Legislation relating to the practice of midwifery, 1931; PRAAD/CSO/5/1/916, Medical practitioners and dentists’ ordinance, 1938–9.

68 See Abena Dove Osseo-Asare, ‘Writing Medical Authority: The Rise of Literate Healers in Ghana, 1930–70’, *The Journal of African History*, 2016, 57, 69–91.

69 See, for details, P. A. Twumasi, ‘A Social History of the Ghanaian Pluralistic Medical System’, *Social Science & Medicine. Medical Anthropology*, 1979, 13B, 349–56; for discussion of epistemic hybridity in the practice of South African science, see Anne Pollock, *Synthesizing Hope: Matter, Knowledge, and Place in South African Drug Discovery* (Chicago: University of Chicago Press, 2019).

70 On Manson and the history of the LSHTM, see Douglas M. Haynes, *Imperial Medicine: Patrick Manson and the Conquest of Tropical Disease* (Philadelphia: University of Pennsylvania Press, 2001).

71 Helen Tilley, *Africa as a Living Laboratory: Empire, Development, and the Problem of Scientific Knowledge, 1870–1950* (Chicago: University of Chicago Press, 2011).

72 The precursor to the ICD was distributed to all colony governments from at least the 1930s, see PRAAD/CSO/11/1/266, ‘Manual of the International List of Causes of Death’, 1931. The ICD is the central example used in Bowker and Starr’s ranging STS analysis of classification, Geoffrey C. Bowker and Susan Leigh Star, *Sorting Things out: Classification and Its Consequences* (Cambridge, MA: MIT Press, 2000).

73 Iliffe, 73; PRAAD/ADM/11/1/1577, Medical and dental scholarship schemes, supervision of scholars in England, 1938–44, P. Z. Ezechiel, ‘Government Scholarships to African Students for the Purpose of Studying Medicine in the United Kingdom’, 1940.

74 Clapperton Chakanetsa Mavhunga, *The Mobile Workshop: The Tsetse Fly and African Knowledge Production* (Cambridge, MA: MIT Press, 2018), xv, 65; although rarely considering science, technology or medicine directly, anti-colonial theorists have long considered the primacy of European languages as leading to the erasure of African epistemologies. See, for instance, Ngũgĩ wa Thiong’o, *Decolonising the Mind: The Politics of Language in African Literature* (Oxford: James Currey, 1986).

75 PRAAD/CSO/11/1/454, Study of Native Medicines, 1938–9.

76
*Jubilee Souvenir*, 93.

77 See, for instance, William Whitla, *Elements of Pharmacy, Materia Medica, and Therapeutics*, 7th edn (London: Henry Renshaw, 1898), viii.

78 Abena Dove Osseo-Asare, *Bitter Roots: The Search for Healing Plants in Africa* (Chicago: The University of Chicago Press, 2014), 131–64.

79 See, for instance, E. A. Badoe, E. Q. Archampong and M. O. A. Jaja, *Principles and Practice of Surgery, Including Pathology in the Tropics* (Tema: Ghana Publishing Corporation, 1986); and the *African Journal of Health Professionals Education* (est. 2009).

80 On this history elsewhere in Ghanaian education provision, see Abena Dove Osseo-Asare, ‘Scientific Equity: Experiments in Laboratory Education in Ghana’, *Isis*, 2013, 104, 713–41; Nkrumah is quoted in C. O. Easmon, ‘Training of Doctors in Ghana’, *Ghana Medical Journal*, 1969, 8, 208–17.

81 UGMS G-52, part 1, ‘Annual and General Reports’, c. 1965–70, Ghana Medical School Progress Report, 1966; UGMS/G-52/2, ‘Annual and General Reports, 1971–87’, University of Ghana Medical School Library, 1966–71.

82 UG/1/3/2/2/129, Medical School Agenda and Minutes of Meetings, 1988–2005, ‘African Perspective on Medical Education: Africa Regional Conference, Brazzaville October 27th-30th, 1987’.

83 PRAAD/RG/3/6/937, University of Ghana Medical School Foreign Exchange Budget, 1985.

84 UG/1/3/2/2/129, ‘Medical School Agenda and Minutes of Meetings, 1988–2005’, University of Ghana Medical School Library Committee Minutes, 3rd March 1987.

85 ‘Homepage | Nuffic’ <https://www.nuffic.nl/en> [accessed 22 April 2022].

86 Osseo-Asare, ‘Scientific Equity’; Noemi Tousignant, ‘Broken Tempos: Of Means and Memory in a Senegalese University Laboratory’, *Social Studies of Science*, 2013, 43, 729–53; Damien Droney, ‘Ironies of Laboratory Work during Ghana’s Second Age of Optimism’, *Cultural Anthropology*, 2014, 29, 363–84.

87 Wendland, 197–200.

88 PRAAD/RG/3/6/993, ‘Inspection of Komfo Anokye Hospital’, 1980.

89 PRAAD/RG/3/6/993, ‘Inspection of Komfo Anokye Hospital’, 1980.

90 On these limitations in North America, see, for instance, Prentice; for an illustration of the huge variety of tasks given to Ugandan medical students in the 1930s, see Hugh Brindley Owen, ‘Medical Education in Uganda’, *Tropical Diseases Bulletin*, 1933, 30, 659–68 (665); quoted in Iliffe, 74–75.

91 RAAD/RG/11/2/82, ‘The Anatomy Act, 1965–6’.

92 At first cadavers were occasionally brought to Tamale from the Police Hospital in Accra and, although UDS now has the capacity to source cadavers locally, there is still no dissection room.

93 This approach to medical training was developed across several North American and European universities during the 1970s and was primarily considered a means to address shortages of General Practitioners. A number of international partners contributed to the development of PBL at UDS, but the curricula which was ultimately adopted was borrowed from Beira, in Mozambique. On the history of PBL, see Virginie F. C. Servant-Miklos, Geoff R. Norman and Henk G. Schmidt, ‘A Short Intellectual History of Problem-Based Learning’, in *The Wiley Handbook of Problem-Based Learning* (Wiley-Blackwell, 2019), 3–24.

94 Patricia Louie and Rima Wilkes, ‘Representations of Race and Skin Tone in Medical Textbook Imagery’, *Social Science & Medicine*, 2018, 202, 38–42.

95 An illustrative example of this relates to the reproduction of historical understandings of gender. See Ericka Johnson, ‘The Ghost of Anatomies Past: Simulating the One-Sex Body in Modern Medical Training’, *Feminist Theory*, 2005, 6, 141–59; Nott and Harris, ‘Sticky models.’

96 On the performance of gynaecology, see Terri Kapsalis, *Public Privates: Performing Gynecology from Both Ends of the Speculum* (Durham, NC: Duke University Press, 1997).

97 For the social history and construction of disease, see, for instance, Charles E. Rosenberg, ‘Disease in History: Frames and Framers’, *The Milbank Quarterly*, 1989, 67, 1–15; Bruno Latour, ‘On the Partial Existence of Existing and Nonexisting Objects’, in Lorraine Daston, ed, *Biographies of Scientific Objects* (Chicago: University of Chicago Press, 2000), 247–69.

98 For these histories, see, for instance, Susanne Klausen, ‘The Race Welfare Society: Eugenics and Birth Control in Johannesburg, 1930–40’, in Saul Dubow, ed, *Science and Society in Southern Africa* (Manchester University Press, 2000), 164–87; Karl Ittmann, *A Problem of Great Importance: Population, Race, and Power in the British Empire, 1918–1973* (Berkeley, CA: University of California Press, 2014).

99 Holly Rose Ashford, ‘Modern Motherhood, Masculinity, and Family Planning in Ghana, 1960–75’, *Journal of West African History*, 2020, 6, 61–91.

100 Andrea M. Bertotti and Skye A. Miner, ‘Constructing Contentious and Noncontentious Facts: How Gynecology Textbooks Create Certainty around Pharma-Contraceptive Safety’, *Social Studies of Science*, 2019, 49, 245–63.

101 Questions around the universality of the ‘demographic transition’ are explored in Caroline Bledsoe, *Contingent Lives: Fertility, Time, and Aging in West Africa* (Chicago, IL: University of Chicago Press, 2002); Jennifer Johnson-Hanks, *Uncertain Honor: Modern Motherhood in an African Crisis* (Chicago, IL: University of Chicago Press, 2006); critiques of the ‘epidemiological transition’ are still developing. See Megan Vaughan, Kafui Adjaye-Gbewonyo and Marissa Mika, eds, *Epidemiological Change and Chronic Disease in Sub-Saharan Africa: Social and Historical Perspectives* (London: UCL Press, 2021).

102 UG 1/3/2/2/129, Medical School Agenda and Minutes of Meetings, 1988–200, ‘University of Ghana Medical School Library Committee Minutes’, 3rd March 1987.

103 For a fuller history of the W.N. Laing Pathology Museum, see Kumoji and Nott.

104 Anthony Amalba and others, ‘The Effect of Community Based Education and Service (COBES) on Medical Graduates’ Choice of Specialty and Willingness to Work in Rural Communities in Ghana’, *BMC Medical Education*, 2016, 16, 79.

105 Easmon, 215.

106 Quoted in Pauline Kusiak, ‘“Tubab” Technologies and “African” Ways of Knowing: Nationalist Techno-politics in Senegal’, *History and Technology*, 2010, 26, 225–49.

107 Kwame Nkrumah, ‘Message to the Ghana Medical Association’, *Ghana Medical Journal*, 1962, 1, 1.

108 Walter D. Mignolo, ‘Epistemic Disobedience, Independent Thought and Decolonial Freedom’, *Theory, Culture & Society*, 2009, 26, 159–81.

109 Warwick Anderson, ‘From Subjugated Knowledge to Conjugated Subjects: Science and Globalisation, or Postcolonial Studies of Science?’, *Postcolonial Studies*, 2009, 12, 389–400 (392).

110 This is a prime example of Simon Choat’s recent argument which, in support of historical materialism in science studies, takes that ‘in its emphasis on relations of becoming, contingency, change, openness and suppleness’ the ‘new materialism’ favoured in STS ‘tends to obscure the very existence of enduring or rigid structures of power and the reproduction of relations of domination and exploitation’. Choat, 1036.

