# Correction to: Material History, Historied Materials and the Question of Epistemic Freedom in Ghana’s Medical Schools

**DOI:** 10.1093/shm/hkad077

**Published:** 2023-09-29

This is a correction to: John Nott, Material History, Historied Materials and the Question of Epistemic Freedom in Ghana’s Medical Schools, Social History of Medicine, 2023;, hkad039, https://doi.org/10.1093/shm/hkad039

The Publisher apologizes for originally publishing a non-copyedited version of this manuscript.

The manuscript has now been corrected as follows: the author’s affiliation information has been updated, references have been corrected throughout the text, a separate Funding section has been added and the Acknowledgements section has been updated.

